# Improving Bystander Self-efficacy to Prevent Violence Against Women Through Interpersonal Communication Using Mobile Phone Entertainment Education: Randomized Controlled Trial

**DOI:** 10.2196/38688

**Published:** 2023-04-14

**Authors:** Ichhya Pant, Bee-Ah Kang, Rajiv Rimal

**Affiliations:** 1 Department of Prevention and Community Health George Washington University Milken Institute School of Public Health Washington, DC United States; 2 Department of Health, Behavior and Society Johns Hopkins University Bloomberg School of Public Health Baltimore, MD United States

**Keywords:** mHealth, voice-response, entertainment education, rural, bystander, self-efficacy, violence against women, interpersonal communication, violence, women, society

## Abstract

**Background:**

Violence against women is a major challenge worldwide and in India. Patriarchal social and gender norms suppress disclosure of violence experienced by women. Stimulating interpersonal communication about a normatively stigmatized but prevalent topic could offer an avenue toward boosting bystander self-efficacy to intervene and prevent violence against women.

**Objective:**

In this study, to reduce violence against women as the distal goal, we adopted a two-pronged strategy grounded in Carey’s model of communication, approaching the issue in an incremental way. First, we aimed to explore whether the intervention promoted interpersonal communication about violence against women as an initial step. Second, we examined whether the intervention improved women’s self-efficacy to intervene when they witness violence in their community through interpersonal communication. Our model is based on the social cognitive theory that posits observational learning (ie, hearing about other women interfering to stop violence) fosters self-efficacy, a proxy for behavior change.

**Methods:**

We conducted a randomized controlled trial of women of reproductive age using a 2-arm study design embedded within a parent trial implemented in Odisha, India. In total, 411 participants were randomly assigned to the violence against women intervention arm or a control arm if they were active mobile phone owners and assigned to the treatment arm of the parent trial. Participants received 13 entertainment education episodes daily as phone calls. The intervention included program-driven, audience-driven, and responsive interaction strategies to facilitate the active engagement of participants. Audience-driven interactions were incorporated throughout the episodes using an interactive voice response system, which allowed participants to like or replay individual episodes through voice-recognition or touch-tone keypad. Our primary analysis involved a structural equation model with interpersonal communication as a potential mediator on the pathway between intervention exposure and bystander self-efficacy to prevent violence against women.

**Results:**

The findings from structural equation modeling demonstrated the significant mediating effect of interpersonal communication on the relationship between program exposure and bystander self-efficacy. Exposure was positively related to interpersonal communication (β=.21, SE=.05; *z*=4.31; *P*<.001) and bystander self-efficacy (β=.19, SE=.05; *z*=3.82; *P*<.001).

**Conclusions:**

Our results demonstrate participant engagement in interpersonal communication following exposure to a “light” entertainment education program with audio-only format via feature phones in rural settings can result in improved self-efficacy to prevent violence against women. This elevates the role of interpersonal communication as a mechanism of behavior change in mobile phone–based interventions, given that most entertainment education interventions tend to be mass media based. Our findings also show the potential of changing the environment where witnesses of violence deem it worthy of intervention and perceive higher efficacy to stop violence in the community, rather than putting the onus on the perpetrator, to prevent any counterproductive effects.

**Trial Registration:**

Clinical Trials Registry-India CTRI/2018/10/016186; https://tinyurl.com/bddp4txc

## Introduction

One in three women worldwide report having experienced violence in their lifetime [1]. In India, this issue is especially pronounced, with half a million cases of violence against women (VAW) reported to the National Criminal Record Bureau in 2019 [2]. The United Nations defines VAW as, “any act of gender-based violence that results in, or is likely to result in physical, sexual or psychological harm or suffering to women, including threats of such acts, coercion or arbitrary deprivation of liberty, whether occurring in public or in private life” [3]. Indian women have reported experiencing physical violence, psychological abuse, or multiple forms of violence [4-8]. Interventions typically focus on periurban and urban localities despite the higher prevalence of VAW in rural areas [9-12]. For instance, television-based interventions such as the *Bell Bajao Campaign* have been effective in raising bystander awareness to counter VAW in urban locales [13]. However, rural India (our study site) offers limited exposure to television campaigns as they are not the preferred way of communication for women [14]. A paucity of interventions implemented and evaluated in resource-constrained settings such as rural India still remains a challenge [15]. VAW often goes undisclosed and remains a taboo social subject due to patriarchal, social, and gender norms, stigmatizing disclosure of VAW [16,17].

When behaviors are both stigmatized and practiced extensively in society, as is the case with VAW in many parts of India [18], one has to be careful about running campaigns that have the potential of further driving the practice underground, as has been observed, for example, in interventions attempting to reduce female genital mutilation [19]. In this project, to reduce VAW as the distal goal, we adopted a two-pronged strategy grounded in Carey’s model of communication [20], approaching the issue in an incremental way. First, we asked whether an intervention could promote interpersonal communication about VAW as an initial step. Carey theorized such exchanges as an *instrumental function* of communication, which occurs when communication serves as a medium to meet existing needs, such as transmission of information between groups (namely, how and why bystanders can and should intervene when they encounter VAW) [20]. Second, we reasoned that, if people could be persuaded to talk about the issue, we would not only keep the issue salient in people’s minds, but we may also improve their self-efficacy to intervene. Some evidence from other domains points to the possibility that this approach could work. For example, study participants engaged in interpersonal communication following intervention exposure appear to improve medication self-efficacy and adherence to iron consumption guidelines in developing and nondeveloping regional settings [21,22]. Following these models, we tested the following hypothesis in a field trial: interpersonal communication will serve as a mediator in the relationship between the intervention and bystander self-efficacy to intervene (H1).

Interpersonal communication, of course, is only 1 (though important) among many pathways that could affect self-efficacy. For example, listening to a story about how others also intervened, the struggles they went through in that decision, and the outcomes they encountered afterward all speak to the idea of observational learning, a key concept in social cognitive theory [23], which can improve self-efficacy [24]. Similarly, observation about others’ behaviors can affect descriptive norms (perceptions about the prevalence of a behavior) [25], which in turn can affect injunctive norms (pressures to conform) and subsequently behavior [26]. For these reasons, in our underlying theory of change, we would also expect a direct pathway that links exposure to the intervention with self-efficacy to intervene, even though we have not articulated these other mechanisms in this paper.

## Methods

### Study Design and Setting

The mobile entertainment education intervention (mRANI) study was part of the parent trial Reduction of Anemia through Normative Innovation (RANI), a cluster randomized study using social norms–based approaches to anemia reduction [27]. We conducted this mRANI study within the RANI treatment arm that received social-norms intervention activities in addition to usual care. mRANI is based on a novel design, which we term the “reciprocal control double intervention” design. In this design, a treatment group for 1 intervention serves as a control group for the other intervention, and vice versa. We randomly assigned participants from the RANI parent trial either to the treatment arm to improve bystander self-efficacy to reduce VAW or to the control arm to improve iron folic acid (IFA) consumption self-efficacy. Thus, for analyses pertaining to IFA consumption, the violence against the women arm serves as the control, and for analyses pertaining to the violence against the women arm (the current study), the IFA consumption arm serves as the control.

Both mRANI arms (VAW and IFA arms) of the study are built on the theory of normative social behavior [28], which postulates that social norms affect behaviors indirectly through various individual and contextual factors, including self-efficacy, risk perception, and gender norms. The theory suggests that descriptive norms (ie, beliefs about the prevalence of a behavior) and injunctive norms (ie, beliefs about the expectation that others have toward oneself) can both affect behaviors. When coupled with self-efficacy, in particular, the relationship between social norms and behaviors can exert greater effects [29,30]. Our study was designed to promote bystander self-efficacy among participants in the treatment arm throughout the norms-based 13-episode of an audio-only entertainment education (EE) program, *Rani Kuhe Kahani*, delivered via phone calls.

Our project was implemented in the Angul district of Odisha, India, where a majority of the residents (83%) live in rural areas and are Hindu (94%) [31]. According to recent estimates, 30% of women in India reported having experienced multiple forms of violence within the past year [4-7,9].

### Patient and Public Involvement Statement

Study participants and expert stakeholders residing in the communities where mRANI was implemented contributed to the design of the intervention. Patients were not involved in this study.

### Randomization and Masking

We used a random number generator to list eligible women in random order who were confirmed to own phones and have operating phone numbers (n=411). Starting with the first woman on a randomly ordered list from the IFA arm, we selected every other woman, and for the VAW arm, we selected every other woman starting with the second woman. Using this method, 205 and 206 women were recruited to be randomized to the treatment arm and the control arm, respectively. Our research team was not masked to group assignment.

### Intervention Design and Delivery

EE has been recognized as an effective health communication strategy to achieve social and behavior change through integrating educational messages into entertainment programs. While EE practice has continued to be diverse in its range of theories, technologies, and health issues, studies have suggested that EE plays a role in stimulating psychosocial and behavioral factors, in turn, to promote health and social change [32-37]. In particular, EE can create an opportunity to expand dialogue at the community level, bolstering the effect of communication interventions [32,35].

We offered an EE program *Rani Kuhe Kahani*, comprised 13 episodes with a storyline of a family’s journey during the COVID-19 pandemic. Each episode lasted approximately 3 to 5 minutes and was delivered daily to participants via phone calls in the afternoons or evenings, depending on their preference. As some participants reported an inability to receive phone calls due to being busy or remained unreachable during the first 13 days of implementation, the entire series was delivered to all participants as a repeat offering. In total, participants had the option of listening to an episode of the program on 4 separate occasions. Intervention delivery occurred between December 2020 and January 2021.

The plot of *Rani Kuhe Kahani* was developed to highlight the continuous rise of VAW in India and encourage our listeners to intervene and prevent VAW within their sphere of influence using norm-based strategies. The program-set descriptive norms around VAW by depicting characters from all walks of life are intervening VAW and incorporated injunctive norms by creating social expectations for the audience to act against VAW. The overall storyline was mirrored for the treatment and control keeping constant the overall length, actors, and sound.

Our program included interactive components at 3 levels: program-driven, audience-driven, and responsive interactions (Textbox 1). The mRANI team inserted program-driven strategies, in the format of jingles, episode teasers, and recaps. Audience-driven interactions were incorporated throughout the episodes using an interactive voice response (IVR) system, which allowed participants to like or replay individual episodes through voice-recognition or touch-tone keypad. The system also enabled mRANI listeners to further engage with the program through responsive interactions. These interactive functions were played at the end of each episode, allowing listeners to take prerecorded quizzes and answer questions about character identification, engagement and satisfaction with the story, and agreement with plot themes.

We further used the IVR system to collect data on process evaluation in real time given the success of prior interventions’ ability to facilitate and track audience engagement using IVR systems [38]. Textbox 2 describes process evaluation indicators collected throughout the mRANI intervention. The IVR system has been found to be particularly effective in rural settings with populations who have limited educational and digital literacy [39,40]. Calls received by participants were free of cost. Details of our program design and delivery can be found in our study protocol [41].

**Mobile entertainment education intervention interactive components.**

**Program-driven interactions**

- Entertainment education program release announcements
- Entertainment education program jingles
- Entertainment education program episode teasers
- Entertainment education program episode recaps

**Audience-driven interactions**

- Like or replay entertainment education episodes on interactive voice response system
- Call mobile entertainment education intervention hotline

**Responsive interactions**

- Quizzes and questions on:
  topically related prosocial themes,
  character identification,
  engagement with the narrative,
  agreement with major plot points,
  satisfaction with the narrative, and
  perception of the most important problem in the village

**Process evaluation indicators for the mobile entertainment education intervention.**

**Dose**

- Number of calls sent
- Number of calls received
- Number of entertainment education episodes heard in full by mobile entertainment education intervention participants

**Audience involvement or engagement**

- Number of interactions with interactive components
- Number of likes on episodes
- Number of replays of episodes
- Number of calls and queries on mobile entertainment education intervention hotline
- Number of referrals to community and clinical linkages

**Attention**

- Number of seconds spent listening to episodes
- Number of seconds spent re-listening to episodes
- Number of seconds spent listening to jingles
- Number of seconds spent listening to episode teasers
- Number of seconds spent listening to episode recaps

**Recall**

- Percentage of participants who accurately recall prosocial themes addressed in the episodes
- Percentage of participants who accurately recall mobile entertainment education intervention characters
- Percentage of participants who accurately recall major mobile entertainment education intervention plot points

**Narrative satisfaction and engagement**

- Percentage of participants who are engaged with the narrative
- Percentage of participants who are satisfied with the narrative

**Character identification**

- Percentage of participants who identify with Malati (protagonist) or Dolly (protagonist’s daughter)

**Most important problem in the village**

- Percentage of participants who identify anemia or violence against women (depending on their assignment) as the most important problem at the end of mobile entertainment education intervention entertainment education program

### Study Procedures

Data collection was conducted via household surveys between February and March 2021, and took approximately 15 minutes of participant time. Demographic characteristics, including age, number of years of education, tribal association, marital status, parity, and digital literacy, were measured. Tribal association was measured with 4 items. We dichotomized women’s tribal association based on whether they reported being part of scheduled tribes, groups of people who make up the most disadvantaged social groups in India. Participants who belonged to the other groups, including scheduled caste, other backward caste, or none, were categorized into nonscheduled tribes. Digital literacy was measured with nine binary items that asked participants’ comfort in using digital technologies and media. Responses were summed into an index variable (α=.76).

“Bystander self-efficacy to prevent VAW” was measured by asking participants the degree to which they agreed with the 8 following items: “If I saw a woman in my community experiencing violence, I am confident I could manage the situation by banging pots and pans (ie, distracting); by striking up a conversation to diffuse the situation (ie, distracting); by checking in on the women after witnessing the violence (ie, delaying); by offering her empathic and supportive words in private afterward (ie, delaying); by recording what is happening on my phone or by taking a picture (ie, documenting); by reporting the incident to local leaders, organizations and authorities to document the incidence (ie, documenting); by seeking help from SHG group members or influential community leaders (ie, delegating); by seeking help from respected elders in the family or the community (ie, delegating).” Responses were recorded on 5-point Likert scales, ranging from “strongly disagree” to “strongly agree” and averaged into an 8-item measure (α=.89).

“Exposure to the Rani Kuhe Kahani program” was measured as the total number of seconds participants spent listening to the EE program and logged objectively by the IVR system.

Lastly, *interpersonal communication* was measured with two items, which asked participants whether they had communicated with others about the program and if they had recommended the program to other women. Response options for both items were recorded as yes or no. Participants were assigned a score of 1 for each positive response, and thus the variable ranged from 0 to 2.

### Ethics Approval

This study was approved by the George Washington University Institutional Review Board (FWA00005945); Sigma Science and Research, an independent institutional review board located in New Delhi, India; and the Indian Council for Medical Research’s Health Ministry’s Screening Committee. The trial has also been registered with the Clinical Trial Registry of India.

#### Participant Orientation, Consent, and Confidentiality

Participants were contacted preintervention delivery to validate their phone numbers, followed by orientation and enrollment. Participants were informed of the objectives and intervention delivery channel and duration. Verbal informed consent was obtained from those who expressed interest in enrolling in the mRANI study. Next, participants noted their preferences for the day and time they prefer to receive the mRANI EE programs. Participants also received contact information for the mRANI hotline set up to answer additional questions and referrals to local supporting agencies.

#### Risk Mitigation Plan

mRANI educational messages were strategically masked within an entertaining narrative tested in the field; thus, we expected minimal sensitivity and negative reactions from our audience or their family members. However, the study team acknowledges that VAW is a sensitive subject matter in Angul, Odisha. The mRANI study, therefore, adhered to the risk mitigation plan in place for the larger RANI trial. Enrolled mRANI participant names and their data were documented in an encrypted file. Confidentiality was maintained by only allowing 1 team member access to these data. Intervention delivery was limited to women who reported being phone owners (rather than sharers or borrowers) to prevent unintended consequences for potential mRANI participants because they have more agency and privacy related to their phone use. Intervention delivery was tailored to the days to ensure their privacy and security. Caller ID clearly identified phone calls associated with the intervention with the name of the mRANI study so there is no ambiguity tied to the source of phone calls or the EE programs. Community and clinical referrals were provided to participants who called the mRANI hotline. Local experts and consultants triaged requests for information and support. Finally, participants had the option to opt out of mRANI EE programs at any time by contacting the mRANI hotline or by pressing 0 on their phone.

### Outcome Measures

Our primary evaluation outcome for this study was bystander self-efficacy to prevent VAW, measured after exposure to the program. We assessed self-efficacy through 8 items, each of which was a statement that asked participants to rate on a 5-point scale (1=strongly disagree; 5=strongly agree) how strongly they agreed with the statement. Representative statements included “If I saw a woman in my community experiencing violence, I am confident I could manage the situation by banging pots and pans,” and “If I saw a woman in my community experiencing violence, I am confident I could manage the situation by striking up a conversation to diffuse the situation.” Responses were averaged into an index (α=.89), such that higher values signified greater efficacy.

### Statistical Analysis

As a first step of data analysis, we calculated frequencies, means, and SDs for all variables of interest by the treatment and the control arm. We then tested correlations among all variables of interest, including demographics, interpersonal communication, exposure to the program, and bystander self-efficacy. A *P* value of less than .05 was deemed statistically significant. Our analysis involved a structural equation model with interpersonal communication as a potential mediator on the pathway between intervention exposure and bystander self-efficacy to prevent VAW. We used Stata for Mac (version 14.2, Statacorp) to conduct all analyses for this study [42].

## Results

Sample characteristics are described in Table 1. Participants in the treatment and control arms showed similar demographic characteristics. On average, the age of participants was 31.42 (SD 8.03) years. Approximately 19% (n=38) of women in the treatment arm and 13% (n=26) of women in the control arm were part of scheduled tribes, respectively. Participants had a mean of 7.73 (SD 3.66) years of education and had a mean of 1.49 (SD 1.00) children on average. On a scale of 0 to 9, participants in the treatment arm had a mean of 3.93 (SD 1.48) and participants in the control arm had a mean of 3.96 (SD 1.58) digital literacy levels.

Table 2 shows the correlation matrix across variables of interest, with the treatment arm below and the control arm above the diagonal. In the treatment arm, the number of children was positively associated with age (*r*=0.47; *P*<.001) and marital status (*r*=0.46; *P*<.001) but negatively associated with education (*r*=−0.36; *P*<.001) and digital literacy (*r*=−0.15; *P*=.04). Bystander self-efficacy was positively associated with both exposure (*r*=0.18; *P*=.01) and interpersonal communication about the program (*r*=0.19; *P*=.009) among participants in the treatment arm. Participants in the control arm showed similar correlation results, as the number of children was positively associated with age (*r*=0.32; *P*<.001) and marital status (*r*=0.51; *P*<.001) but negatively associated with education (*r*=−0.26; *P*<.001) and digital literacy (*r*=−0.19; *P*=.007). Digital literacy was positively associated with education in both arms (*r*=0.36, *P*<.001 for treatment; *r*=0.41, *P*<.001 for control). Bystander self-efficacy was significantly correlated with education (*r*=0.20; *P*=.005) and exposure (*r*=0.15; *P*=.04), but not with interpersonal communication in the control arm. Interpersonal communication was positively associated with exposure in the treatment arm (*r*=0.39; *P*<.001) as well as in the control arm (*r*=0.41; *P*<.001).

**Figure 1:**
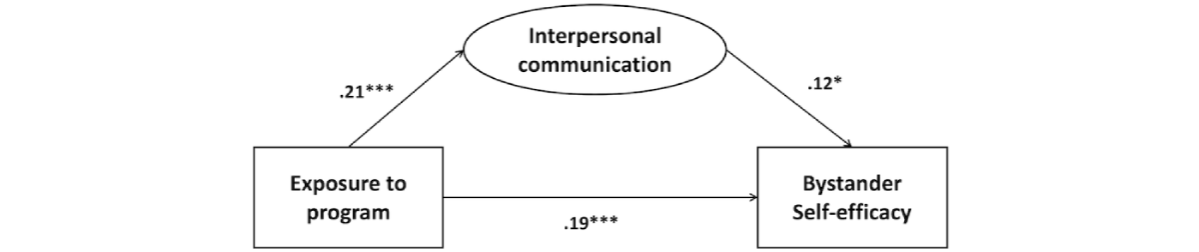
Path coefficients in the structural equation model. *P<.05; **P<.01; ***P<.001.

The findings from structural equation modeling demonstrated the significant mediating effect of interpersonal communication on the relationship between program exposure and bystander self-efficacy (Figure 1). Exposure was positively associated with interpersonal communication (β=.21, SE=.05; *z*=4.31; *P*<.001) and bystander self-efficacy (β=.19, SE=.05; *z*=3.82; *P*<.001). The relationship between exposure and bystander self-efficacy was partially mediated by interpersonal communication. The path between the mediator (ie, interpersonal communication), and bystander self-efficacy was significant (β=.12, SE=.05; *z*=2.30; *P*=.02).

**Table 1:** Characteristics of the sample in treatment and control arms (N=411).

Participant characteristics			Total (N=411)		Treatment (n=205)	Control (n=206)
Age (years), mean (SD)			31.42 (8.03)		31.54 (8.39)	31.30 (7.67)
**Tribe, n (%)**						
	Scheduled tribe^a^	64 (16.12)		38 (18.91)		26 (13.27)
	Nonscheduled tribe	333 (83.88)		163 (81.09)		170 (86.73)
Education (years), mean (SD)			7.73 (3.66)		7.69 (3.65)	7.76 (3.67)
**Marital status, n (%)**						
	Married	334 (84.13)		168 (83.58)		166 (84.69)
	Not married	63 (15.87)		33 (16.42)		30 (15.31)
Number of children, mean (SD)			1.49 (1.00)		1.47 (1.03)	1.52 (0.96)
Digital literacy, mean (SD)			3.95 (1.53)		3.93 (1.48)	3.96 (1.58)

^a^Scheduled tribes are groups of people who make up the most disadvantaged social groups and have low socioeconomic status in India.

**Table 2:** Zero-order Pearson correlations among key variables in treatment and control arms (N=411)^a^.

Treatment and control			Age		Caste		Education		Marital status		Number of children		Digital literacy		Exposure		Interpersonal communication		Bystander self-efficacy
**Age**																			
	*r*	1		−0.15		−0.36		0.07		0.32		0.33		0.10		0.08		−0.10	
	*P* value	—^b^		.03		<.001		.37		<.001		<.001		.16		.26		.17	
**Caste**																			
	*r*	−0.03		1		−0.11		−0.13		−0.10		−0.05		−0.01		−0.09		0.00	
	*P* value	.65		—		.13		.08		.16		.46		.90		.21		.98	
**Education**																			
	*r*	−0.50		−0.10		1		0.05		−0.26		0.41		0.05		0.13		0.20	
	*P* value	<.001		.16		—		.50		<.001		<.001		.51		.07		.005	
**Marital status**																			
	*r*	0.11		0.01		−0.03		1		0.51		−0.06		0.02		−0.03		0.11	
	*P* value	.15		.91		.69		—		<.001		.40		.77		.71		.14	
**Number of children**																			
	*r*	0.47		−0.01		−0.36		0.46		1		−0.19		−0.00		0.02		0.03	
	*P* value	<.001		.89		<.001		<.001		—		.007		.97		.77		.73	
**Digital literacy**																			
	*r*	−0.22		−0.00		0.36		0.00		−0.15		1		−0.05		0.04		0.13	
	*P* value	.002		.99		<.001		.96		.04		—		.52		.56		.08	
**Exposure**																			
	*r*	0.11		−0.13		0.06		0.09		0.14		−0.04		1		0.41		0.15	
	*P* value	.14		.08		.40		.18		.06		.62		—		<.001		.04	
**Interpersonal communication**																			
	*r*	−0.11		−0.03		0.16		−0.08		−0.11		−0.02		0.39		1		0.11	
	*P* value	.12		.66		.02		.26		.14		.74		<.001		—		.12	
**Bystander self-efficacy**																			
	*r*	−0.01		−0.00		0.09		0.01		−0.01		−0.18		0.18		0.19		1	
	*P* value	.88		.95		.20		.88		.85		.01		.01		.01		—	

^a^Correlations in the treatment arm are below the diagonal and those in the control arm are above the diagonal.

^b^Not applicable.

## Discussion

### Principal Findings

The primary goals of this study were to determine whether an EE-based intervention could enhance people’s self-efficacy to intervene when they witness acts of VAW and, if so, to determine whether interpersonal communication can serve as the underlying mechanism for this change. The answers to both questions seem to be affirmative, though with a modest magnitude of impact. Consistent with our expectations, those who listened to the intervention had higher levels of self-efficacy to intervene, a significant (though not exclusive) portion of the effect being mediated through interpersonal communication.

Thus, data from this field trial indicate that interventions can contribute to the chain of events, whereby interventions affect interpersonal communication that in turn affects self-efficacy to intervene (which may further improve bystander interventions). We found that interpersonal communication served as a partial mediator in the relationship between intervention exposure and self-efficacy. The partial mediation suggests, of course, that other factors were also at play in how the intervention affected self-efficacy, something beyond the scope of this study to pursue, although we hope other researchers can take this on. We also observed a direct impact pathway from the intervention to self-efficacy (above and beyond its effect through interpersonal communication). This further suggests the importance of investigating other factors that may have played an important role, something we defer to future research.

For several reasons, we consider the findings from this paper to represent a first, though important, step-in efforts to reduce VAW. First, we assume that, for potentially stigmatizing (though prevalent) behaviors, it is better to adopt an incremental approach rather than a heavy-handed and intense one that can lead to counterproductive effects [43]. Second, even though the ultimately desired outcome is to put the onus on the perpetrator not to engage in the violence, we sought a different route: to change the environment where witnesses of violence deem it worthy of intervention and perceive higher efficacy to do so. Doing so, we reasoned, was a critical factor in changing social norms around VAW, to make violence both less prevalent (lower descriptive norms) and less acceptable (lower injunctive norms). Finally, we also assumed that the efficacy to intervene could be affected by an intervention that promoted interpersonal communication about VAW, to keep the issue salient in people’s minds.

Another noteworthy aspect of this study was that the intervention itself was rather “light,” in that it was delivered without visual cues, in audio-only format, and with the intervention messages embedded deep within an entertainment program that spanned 13 episodes. It is difficult to tell whether this low dose was a virtue or a vice. It may have appeared in people’s consciousness in a subtle manner, which would reduce their counterarguments (as has been reported in other studies [44]) and suppress message resistance (as compared to, say, exposure to the message in an overtly persuasive manner). This suggests that the low dose worked in our favor. On the other hand, the relatively small effect sizes we observed in this study could have been the results of the nature of the intervention: its dose was subtle and mild, embedded within an entertainment program delivered only on the phone. We suspect it was the latter, given the “noisy” environment in which messages were likely being processed. Rural women in India tend to be extremely busy and occupied with extensive household chores [45], thus depriving them of opportunities to take time off to listen at length and under undistracted conditions to a program such as ours.

To our knowledge, this is the first randomized field trial that uses cell phone recordings as vehicles to promote bystander interventions. It not only adds to the EE literature but also elevates the role of interpersonal communication as a mechanism of change, even though most EE interventions tend to be mass media based [27]. Another strength of this study is the use of the novel reciprocal control double intervention design, whereby 2 interventions can be run simultaneously, with 1 using the other as a frame of reference. We hope other researchers will adopt this design and pressure test its internal and external validity.

The extant literature on EE is extensive, some focusing on theoretical explanations for its effects and others investigating the underlying theoretical propositions [46,47]. In its broadest sense, EE has involved strategically inserting drivers of behavior change into programs whose primary function is to entertain. Those drivers often involve visual cues, plot lines, and other mechanisms that do not detract from the entertainment value of the program. To that extent, our intervention was no different: we presented a narrative story that included bystander behaviors at strategically chosen points in the script. What is different in this study is the elevated role of interpersonal communication to determine whether our program was entertaining enough that it would be the topic of conversation and, if that were the case, such discussions would result in heightened efficacy. Answers to both questions seem to be a modest yes.

Another manner in which this study contributes to the extant literature is its documented link between interpersonal communication and self-efficacy. In social cognitive theory, Bandura [23] lays out the four sources of self-efficacy: performance accomplishment, affective arousal, observational learning, and verbal persuasion. We suspect that observational learning was likely one of the sources of efficacy enhancement: hearing about other women interfering to stop a violent act in progress could have communicated to our participants that they could do so, too.

### Limitations

This study also includes a number of limitations, the primary one of which pertains to the external validity of the study findings. Women who participated in this study were all part of the parent RANI trial, and the study itself was conducted toward the end of the trial. Participants were thus aware of their own prior participation, as they had already been interviewed for the baseline and the midline data collection efforts. They were likely invested in the study in some way, which may have led them to have a greater commitment to listen to the program. Because both arms of this study were embedded in the treatment arm of the parent trial, this commitment likely applied to both arms of this study.

Another limitation pertains to our observation that interpersonal communication was only a partial mediator of improved self-efficacy, implying that there were other unmeasured variables that likely played a role as well. We also observed that women in the treatment arm were more likely to recommend the program to others; unfortunately, we cannot tell the extent to which this is due to their own stronger response to the program, as opposed to the heightened levels of interpersonal communication (as our theory of change would hypothesize).

We also note that efficacy to intervene is different from intentions or actually intervening, when many other variables could play a part in the decision to intervene, including fear for one’s safety, not wanting to stand out in the crowd, and so forth. Nevertheless, given the literature on the relationship between self-efficacy to intervene and intervening in bullying [48] and other violent behaviors [49], we believe our findings speak to a necessary first step of the underlying psychosocial change that is required.

## Appendix

Multimedia Appendix 1 CONSORT checklist.

## Abbreviations

EE: entertainment education
IFA: iron folic acid
IVR: interactive voice response
mRANI: mobile entertainment education intervention
RANI: Reduction of Anemia through Normative Innovation
TNSB: theory of normative social behavior
VAW: violence against women

## Data Availability

RANI project data and a data dictionary is publicly available since 2022 [50].

## References

[1] (2013). Global and regional estimates of violence against women: prevalence and health effects of intimate partner violence and non-partner sexual violence. World Health Organization.

[2] (2020). Crime in India. Indian National Crime Record Bureau.

[3] Melander G, Alfredsson G, Holmstrom L (2004). Declaration on the elimination of violence against women. The Raoul Wallenberg Institute Compilation of Human Rights Instruments.

[4] Kalokhe AS, Potdar RR, Stephenson R, Dunkle KL, Paranjape A, Del Rio C, Sahay S (2015). How well does the World Health Organization definition of domestic violence work for India?. PLoS One.

[5] Silverman JG, Balaiah D, Decker MR, Boyce SC, Ritter J, Naik DD, Nair S, Saggurti N, Raj A (2016). Family violence and maltreatment of women during the perinatal period: associations with infant morbidity in Indian slum communities. Matern Child Health J.

[6] Raj A, McDougal L (2014). Sexual violence and rape in India. Lancet.

[7] Kalokhe A, Del Rio C, Dunkle K, Stephenson R, Metheny N, Paranjape A, Sahay S (2017). Domestic violence against women in India: a systematic review of a decade of quantitative studies. Glob Public Health.

[8] Daruwalla N, Machchhar U, Pantvaidya S, D'Souza V, Gram L, Copas A, Osrin D (2019). Community interventions to prevent violence against women and girls in informal settlements in Mumbai: the SNEHA-TARA pragmatic cluster randomised controlled trial. Trials.

[9] Daruwalla N, Jaswal S, Fernandes P, Pinto P, Hate K, Ambavkar G, Kakad B, Gram L, Osrin D (2019). A theory of change for community interventions to prevent domestic violence against women and girls in Mumbai, India. Wellcome Open Res.

[10] Krishnan S, Subbiah K, Khanum S, Chandra PS, Padian NS (2012). An intergenerational women's empowerment intervention to mitigate domestic violence: results of a pilot study in Bengaluru, India. Violence Against Women.

[11] Jejeebhoy SJ, Santhya KG (2018). Preventing violence against women and girls in Bihar: challenges for implementation and evaluation. Reprod Health Matters.

[12] More NS, Das S, Bapat U, Alcock G, Manjrekar S, Kamble V, Sawant R, Shende S, Daruwalla N, Pantvaidya S, Osrin D (2017). Community resource centres to improve the health of women and children in informal settlements in Mumbai: a cluster-randomised, controlled trial. Lancet Glob Health.

[13] Balsarkar G (2021). Let us ring the bell on domestic violence…. call for ceasefire. J Obstet Gynaecol India.

[14] Pant I, Rimal RN, Yilma H Not all women are equal in their digital inequality:mHealth interventions informed by the digital divide Virtual poster presentation. Annual Meeting of the American Public Health Association 2020.

[15] Nair N, Daruwalla N, Osrin D, Rath S, Gagrai S, Sahu R, Pradhan H, De M, Ambavkar G, Das N, Dungdung GP, Mohan D, Munda B, Singh V, Tripathy P, Prost A (2020). Community mobilisation to prevent violence against women and girls in eastern India through participatory learning and action with women's groups facilitated by accredited social health activists: a before-and-after pilot study. BMC Int Health Hum Rights.

[16] (2017). Tackling violence against women: a study of state intervention measures (a comparative study of impact of new laws, crime rate and reporting rate, change in awareness level). Bharatiya Stree Shakti.

[17] (2012). Changing cultural and social norms that support violence. Series of briefings on violence prevention: the evidence. World Health Organization.

[18] Palermo T, Bleck J, Peterman A (2014). Tip of the iceberg: reporting and gender-based violence in developing countries. Am J Epidemiol.

[19] Muzima L (2016). Towards a sensitive approach to ending female genital mutilation/cutting in Africa. SOAS L J.

[20] Carey JW (2008). Communication as Culture: Essays on Media and Society.

[21] Archiopoli A, Ginossar T, Wilcox B, Avila M, Hill R, Oetzel J (2016). Factors of interpersonal communication and behavioral health on medication self-efficacy and medication adherence. AIDS Care.

[22] Ganjoo R, Rimal RN, Talegawkar SA, Sedlander E, Pant I, Bingenheimer JB, Chandarana S, Aluc A, Jin Y, Yilma H, Panda B (2021). Improving iron folic acid consumption through interpersonal communication: findings from the reduction in anemia through normative innovations (RANI) project. Patient Educ Couns.

[23] Bandura A (1986). Social foundations of thought and action. Theories in Health Psychology.

[24] Zulkosky K (2009). Sel-efficacy: a concept analysis. Nurs forum.

[25] Kallgren CA, Reno RR, Cialdini RB (2000). A focus theory of normative conduct: when norms do and do not affect behavior. Pers Soc Psychol Bull.

[26] Rimal RN (2008). Modeling the relationship between descriptive norms and behaviors: a test and extension of the theory of normative social behavior (TNSB). Health Commun.

[27] Yilma H, Sedlander E, Rimal RN, Pant I, Munjral A, Mohanty S (2020). The reduction in anemia through normative innovations (RANI) project: study protocol for a cluster randomized controlled trial in Odisha, India. BMC Public Health.

[28] Rimal RN, Real K (2016). How behaviors are influenced by perceived norms. Commun Res.

[29] Jain P, Humienny R (2020). Normative influences on the role of prescription medicine misuse among college students in the United States. Health Commun.

[30] Jang SA, Rimal RN, Cho N (2013). Normative influences and alcohol consumption: the role of drinking refusal self-efficacy. Health Commun.

[31] Government of India (2011). Census of India: 2011.

[32] Storey D, Sood S (2013). Increasing equity, affirming the power of narrative and expanding dialogue: the evolution of entertainment education over two decades. Critical Arts.

[33] Riley AH, Sood S, Mazumdar PD, Choudary NN, Malhotra A, Sahba N (2017). Encoded exposure and social norms in entertainment-education. J Health Commun.

[34] Bae H (2008). Entertainment-education and recruitment of cornea donors: the role of emotion and issue involvement. J Health Commun.

[35] Creel AH, Rimal RN, Mkandawire G, Böse K, Brown JW (2011). Effects of a mass media intervention on HIV-related stigma: 'radio diaries' program in Malawi. Health Educ Res.

[36] Rimal RN, Creel AH (2008). Applying social marketing principles to understand the effects of the radio diaries program in reducing HIV/AIDS stigma in Malawi. Health Mark Q.

[37] Shen F, Han J (2014). Effectiveness of entertainment education in communicating health information: a systematic review. Asian J Commun.

[38] Wang H, Singhal A (2017). Unfurling the Voicebook of Main Kuch Bhi Kar Sakti Hoon: Real-time Audience Engagement, Rising Fandom, and Spurring of Prosocial Actions.

[39] Crawford AG, Sikirica V, Goldfarb N, Popiel RG, Patel M, Wang C, Chu JB, Nash DB (2005). Interactive voice response reminder effects on preventive service utilization. Am J Med Qual.

[40] Kassavou A, Sutton S (2018). Automated telecommunication interventions to promote adherence to cardio-metabolic medications: meta-analysis of effectiveness and meta-regression of behaviour change techniques. Health Psychol Rev.

[41] Pant I, Rimal R, Yilma H, Bingenheimer J, Sedlander E, Behera S (2021). mHealth for anemia reduction: protocol for an entertainment education-based dual intervention. JMIR Res Protoc.

[42] (2018). STATA Corporation.

[43] Salmon CT, Byrne S, Fernandez L, Arval J, Rivers L (2013). Exploring unintended consequences of risk communication messages. Effective Risk Communication.

[44] Tormala ZL, Petty RE (2004). Resisting persuasion and attitude certainty: a meta-cognitive analysis. Resist Persuasion.

[45] Sedlander E, Rimal RN (2019). Beyond individual-level theorizing in social norms research: how collective norms and media access affect adolescents' use of contraception. J Adolesc Health.

[46] Singhal A, Cody MJ, Rogers EM, Sabido M (2003). Entertainment-education and social change. History, Research, and Practice.

[47] Sood S, Menard T, Witte K (2003). The theory behind entertainment-education. Entertainment-Education and Social Change.

[48] Thornberg R, Jungert T (2013). Bystander behavior in bullying situations: basic moral sensitivity, moral disengagement and defender self-efficacy. J Adolesc.

[49] Sjögren B, Thornberg R, Wänström L, Gini G (2021). Bystander behaviour in peer victimisation: moral disengagement, defender self-efficacy and student-teacher relationship quality. Res Pap Educ.

[50] The RANI Project Full Dataset. Figshare.

